# Association of multiple splanchnic venous thrombosis and left renal venous thrombosis, a rare complication of pancreatitis: a case report

**DOI:** 10.1186/s13256-019-2053-4

**Published:** 2019-06-04

**Authors:** Youssef Motiaa, Zakaria Ouassou, Houda Moumou, Wafae el Otmani

**Affiliations:** 1 Intensive Care Unit, Hassan I Hospital, Tiznit, Morocco; 2 Department of Anesthesiology and Intensive Care, Mohammed V Military Hospital, Faculty of Medicine and Pharmacy, Mohamed V University, Rabat, Morocco; 3 Department of Radiology, Hassan I Hospital, Tiznit, Morocco; 4 Cardiac ICU, Mohamed V Military Hospital, Rabat, Morocco

**Keywords:** Splanchnic venous thrombosis, Left renal vein thrombosis, Pancreatitis

## Abstract

**Background:**

Vascular complications of acute pancreatitis are common. Splanchnic thrombosis accounts for 11% of these complications, whereas extrasplanchnic thrombosis remains a rare entity. These complications are associated with high morbidity and mortality. Diagnosis is established on the basis of clinical and radiological evaluation, especially computed tomography. Renal vein thrombosis has been reported previously, but only in association with thrombosis of the inferior vena cava. To our knowledge, renal vein thrombosis without inferior vena cava thrombosis has never been reported in the literature. We report a case of a woman who developed acute pancreatitis complicated with splanchnic thrombosis and renal vein thrombosis with a patent inferior vena cava.

**Case presentation:**

A 48-year-old Moroccan woman with no significant past medical history presented to our emergency department with worsening epigastric pain and vomiting. Her physical examination was notable only for moderate epigastric tenderness. She was apyrexic and had no jaundice or any features of liver failure. An initial computed tomographic scan showed Balthazar grade C pancreatitis with multiple splanchnic thromboses involving the portal vein, superior mesenteric vein, and left renal vein and enteromesenteric venous infarct with no signs of bowel perforation. The inferior vena cava was patent. Therapeutic anticoagulation and analgesia were started with resumption of enteral feeding 72 h later. The result of a thrombophilia screen was negative. Two months later, the patient was admitted to the intensive care unit with acute liver failure. Computed tomography of the abdomen showed worsening ischemic liver lesions and no signs of bowel perforation. Biochemical analysis showed acute hepatitis with hepatocellular insufficiency. The clinical evolution was unfavorable, and the patient died 48 h later.

**Conclusions:**

Association of splanchnic and renal vein thrombosis without inferior vena cava thrombosis as a complication of acute pancreatitis has never been reported before. There are no specific aspects of management of this complication; therapeutic anticoagulation and symptomatic treatment are the main measures used owing to the lack of available organs for liver transplant. The prognosis depends on the consequences of splanchnic thrombosis and their complications.

**Electronic supplementary material:**

The online version of this article (https://doi.org/10.1186/s13256-019-2053-4) contains supplementary material, which is available to authorized users.

## Background

Pancreatitis is an inflammatory disease of the pancreas that can be either acute or chronic and can lead to local or systemic complications [1]. Deep venous thrombosis (DVT), pulmonary embolism, and splanchnic thrombosis have been reported in the literature as complications of pancreatitis [2]. Vascular complications during pancreatitis are a major cause of morbidity and mortality [3]. Almost 25% of patients with pancreatitis develop vascular complications, 11.4% of which are venous splanchnic thrombosis involving the portal vein, splenic vein, and superior mesenteric vein [4].

Extrasplanchnic thrombotic complications have been described in the literature. They involve both venous and arterial circulations: pulmonary circulation [5], inferior vena cava (IVC) and renal veins [6, 7], right atrium [8], coeliac axis [9], and renal arteries [10].

We report a fatal evolution of association of left renal thrombosis and multiple splanchnic venous thrombosis complicating acute pancreatitis in a 48-year-old woman. To our knowledge, renal vein thrombosis in the absence of IVC thrombosis has never been in reported. Written consent to publish our patient’s case information was obtained from the patient’s next of kin.

## Case presentation

A 48-year-old gravida 5 para 5 Moroccan woman with no significant past medical history, including no personal history of thrombophilia or recent surgery and no family history of thromboembolic events or autoimmune disease, presented to our emergency department with a 10-day history of epigastric pain radiating to the back and vomiting. Clinical examination revealed epigastric tenderness. The patient was apyrexic with no jaundice or clinical features of hepatic failure. She was hemodynamically stable; her visual analogue scale score was between 6 and 8; she was conscious with a Glasgow Coma Scale score of 15; she had no hemorrhagic manifestations; and she denied drug intake or alcohol consumption. The result of a urine dipstick test was negative for blood and protein. Biological investigation revealed an elevated lipase level (600 IU/L). Her C-reactive protein level was 28 mg/L. The rest of the blood test results were within normal range, including renal function, hepatic function tests, and coagulation. Her platelet count was 240,000/mm^3^.

**Fig. 1:**
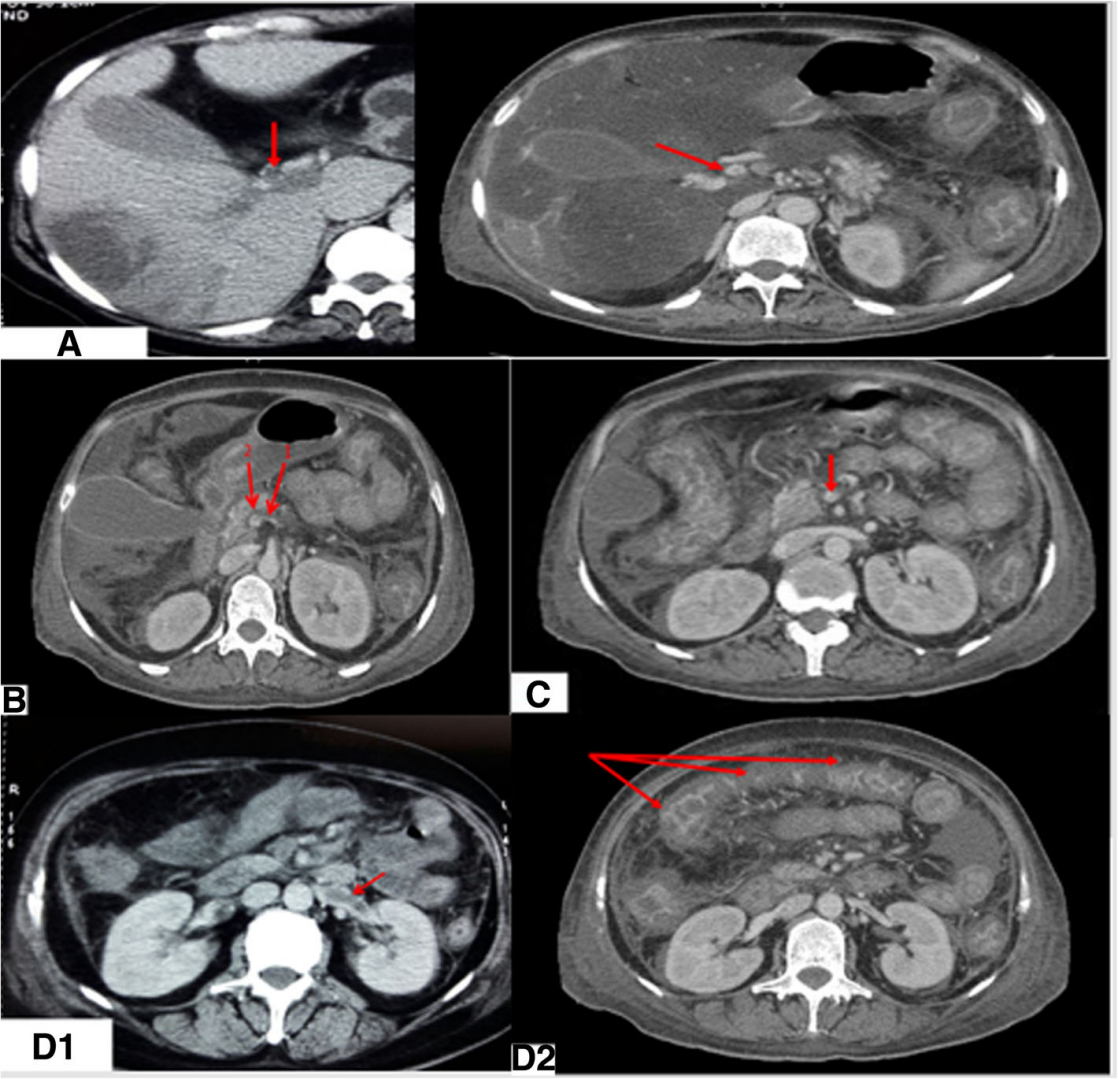
Balthazar grade C pancreatitis with multiple venous thrombosis. The arrows are pointing to: **a** portal venous thrombosis, **b** splenomesareic trunk thrombosis: 1: splenic vein, 2: Superior mesenteric vein. **c** superior mesenteric vein thrombosis. **d** D1: left renal vein thrombosis, D2: mesenteric infarct

**Fig. 2:**
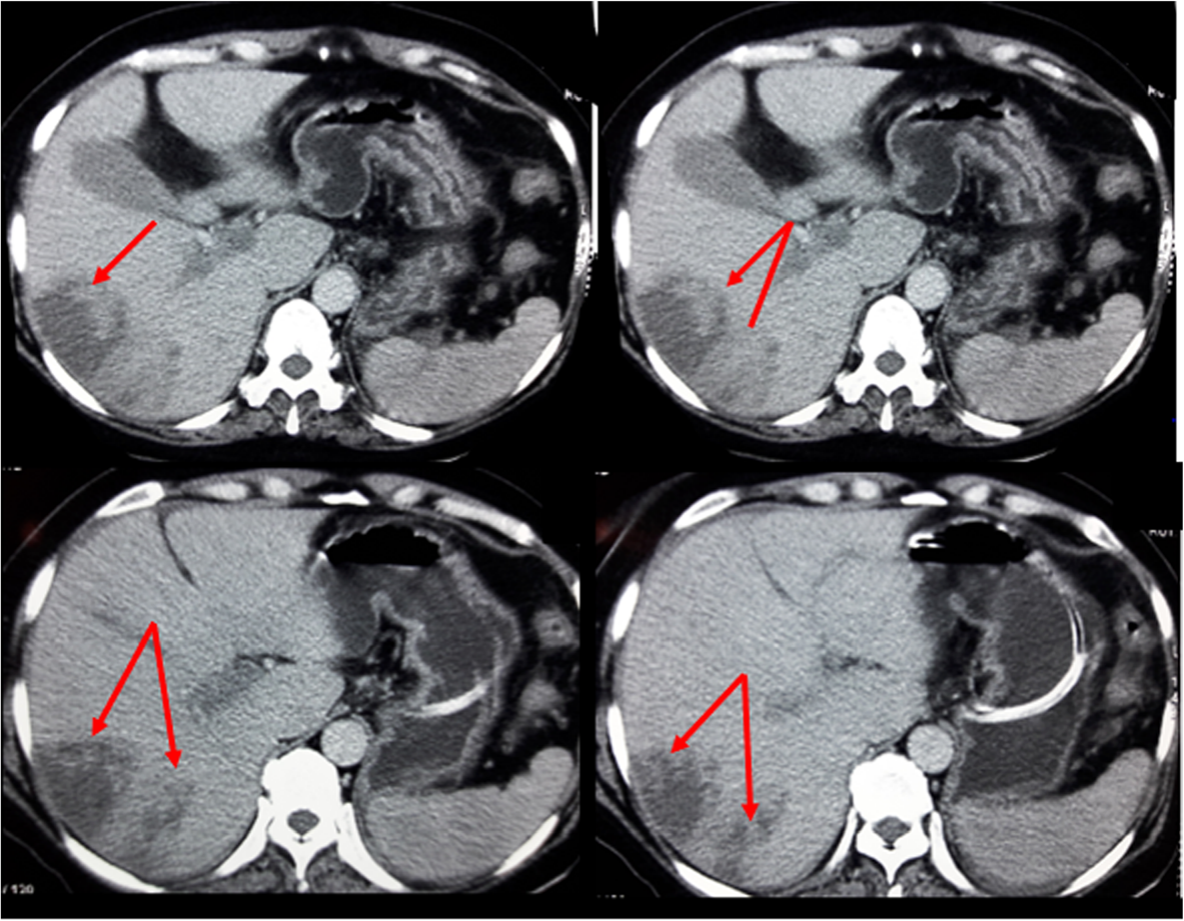
Arrows show ischemic liver lesions

Contrast-enhanced computed tomography (CECT) of the abdomen was performed, which revealed Balthazar grade C pancreatitis with multiple splanchnic thromboses involving the portal vein, superior mesenteric vein, and left renal vein and enteromesenteric venous infarct with no signs of bowel perforation. No free intraperitoneal fluid was observed (Fig. 1). The patient had multiple liver lesions with double components: isodense and hypodense lesions in segments V, VI, and VII and a hypodense lesion in segment VIII of ischemic origin (Fig. 2). Lower limb venous Doppler sonography ruled out DVT.

The result of a thrombophilia screen was negative. Anti-DNA, antinuclear, and anticardiolipin antibodies; anti-β_2_-glycoprotein 1; and anti-factor II were all negative. Functional activity of antithrombin III, protein C, and protein S were 79%, 80%, and 74.5%, respectively. Viral serology results were negative as well.

Therapeutic anticoagulation was started using enoxaparin 1 mg/kg twice daily. The patient had a nasogastric tube inserted for 72 h. She was started on proton pump inhibitors and was kept nil by mouth until the vomiting settled, then oral feeding was gradually introduced. For analgesia, we used regular paracetamol 3 g daily and nefopam 100 mg daily. The evolution was favorable, and the patient was discharged 1 week later to the care of the gastroenterology team.

**Fig. 3:**
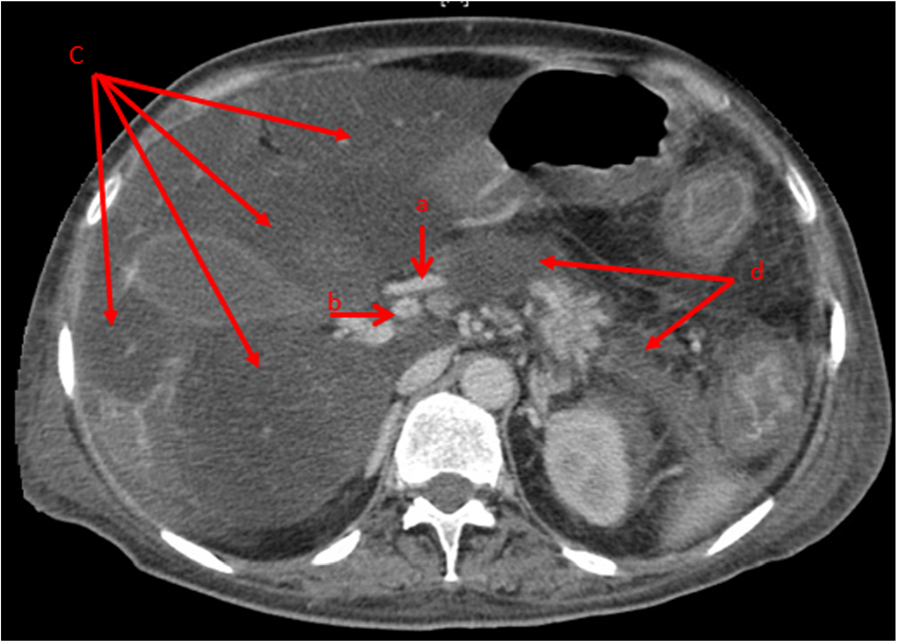
Worsening of liver lesions without bowel perforation. The arrows are pointing to: **a** patent hepatic artery, **b** portal venous thrombosis, **c** hepatic ischemia, **d** pancreatic necrosis

Two months later, the patient was readmitted to the intensive care unit with a 4-day history of confusion. Physical examination revealed jaundice, mild tachycardia with normal blood pressure, and hypoglycemia 0.4 g/L. The patient’s abdomen was distended, tense, and tender to palpation with ascites. Computed tomography (CT) of the abdomen revealed persistent splanchnic and renal thrombosis and worsening of hepatic lesions with extension of bowel ischemia. The hepatic artery was patent, and there were no signs of necrosis or bowel perforation (Fig. 3). Prothrombin time was 25%, aspartate aminotransferase was 10 times normal, alanine aminotransferase was 8 times normal, and total bilirubin was 180 mg/L. Repeat viral serology results were all negative.

Anticoagulation was stopped, and therapeutic measures for hepatic failure were initiated. The patient deteriorated to multiple organ failure with grade 4 hepatic encephalopathy, and she died 48 h later. The patient’s history, symptoms, diagnostic and management are summarized in Additional file 1.

## Discussion

Left renal venous thrombosis can complicate acute pancreatitis even in the absence of IVC thrombosis. This complication can be associated with splanchnic thrombosis. Approximately one-fourth of patients with acute pancreatitis may develop vascular complications. The most common are venous thrombosis, hemorrhage by erosion or rupture of false pancreatic and peripancreatic aneurysms, and formation of varices or bleeding into a pseudocyst. Venous thrombosis is less frequent than hemorrhagic complications; its incidence is 1–2% [11]. The splanchnic circulation is most often affected during pancreatitis. Extrasplanchnic thromboses have been reported, but renal vein thrombosis in the absence of IVC thrombosis has never been reported in the literature, to our knowledge.

The pathophysiology of splanchnic venous thrombosis is related to the close proximity to the pancreas. Many factors can lead to splanchnic venous thrombosis:

- Edema, cellular infiltration, and inflammation are processes that can involve the vein and cause intima injury.
- Vein compression by a pseudocyst or enlarged pancreas leads to impaired venous drainage.
- The elevation of mediators of inflammation, such as tumor necrosis factor, interleukin (IL)-1β, and IL-6, is responsible for activating the hemostatic process.
- Direct exposition of the pancreatic tissue factor can activate the coagulation cascade [2].

In addition to these local reactions, some studies have shown that in acute pancreatitis, mean platelet volume, fibrinogen level, and D-dimer level are high, which increases the tendency to platelet aggregation, disruption of pancreatic microcirculation, and hypercoagulability [12].

Clinically, splanchnic thrombosis may be asymptomatic. In clinical practice, it may be difficult to distinguish between pain due to pancreatitis and pain due to splanchnic thrombosis. In addition to pain, other manifestations of splanchnic thrombosis can be abdominal mass due to splenomegaly, hemorrhage due to portal hypertension, or signs of hypersplenism [2]. The symptoms reported by our patient were gastrointestinal signs such as epigastric pain, nausea, and vomiting. The persistence of portal thrombosis led to severe portal hypertension and hepatic failure with hypoglycemia, ascites, and encephalopathy. The diagnosis was confirmed by CECT of the abdomen, which remains the best way to assess the severity of pancreatitis; detect almost all major complications; and help study vascular structures, intestinal wall, and mesentery with a sensitivity of almost 90% [13].

Venous Doppler sonography of the patient’s lower limbs ruled out DVT. A thrombophilia screen was performed in the context of unusual site of thrombosis as recommended by some authors [14]. In our patient’s case, the renal venous thrombosis occurred even though the IVC was patent.

Anticoagulation in patients with splanchnic thrombosis, according to some authors, should be considered if there is no gastrointestinal bleeding or once the bleeding is controlled and the patient is stable. In this clinical situation, therapeutic anticoagulation is associated with better overall survival, lower risk of recurrence, and better repermeabilization, but the risk of bleeding remains higher. The recommended anticoagulant treatment duration is 3 to 6 months of low-molecular-weight heparin; dosage should be reduced in case of thrombocytopenia [15].

For our patient, we started subcutaneous enoxaparin 1 mg/kg twice daily in the absence of gastrointestinal bleeding; anti-vitamin K anticoagulant was not started, and the patient received symptomatic treatment of pancreatitis.

In acute pancreatitis, inflammation can cause damage to the vessels and lead to hepatic infarcts. However, infarction remains a rare complication of portal vein thrombosis because of the arterial compensation [16]. In our patient, several factors contributed to the unfavorable outcome: idiopathic cause of pancreatitis, which is an independent risk factor of mortality [16]; the absence of splanchnic venous thrombus regression; and the extension of venous intestinal ischemia despite anticoagulation. The clinical and biological evolution was unfavorable in the absence of possibility of liver transplant in our context.

Indication for thrombolysis, either directly with percutaneous transhepatic or transjugular intrahepatic or indirectly by injecting the thrombolytic agent into the superior mesenteric artery, or even endovascular embolectomy, could have been interesting alternatives for our patient, in view of rapid clinical deterioration and persistence of vascular thrombosis, but they are not available in our context [2]. The renal vein thrombosis was nonocclusive and did not cause any specific symptoms. This site of thrombosis, in absence of IVC thrombosis, has never been reported in the literature.

## Conclusions

Splanchnic venous thrombosis is a well-known complication of pancreatitis. It can be asymptomatic sometimes, but it can be responsible for higher mortality. Necessary investigations should be performed when pancreatitis is suspected. CT of the abdomen is used to assess the severity of pancreatitis, to detect local and vascular complications, and to assess their extrasplanchnic extension. Early anticoagulant treatment in absence of contraindication can reduce mortality and recurrence of thromboses and can improve repermeabilization. The association of an extrasplanchnic localization does not change the management in absence of specific symptoms.

## Additional file

Additional file 1: Relevant Past Medical History and Interventions (DOCX 15 kb)

## References

[1] Agarwal N, Pitchumoni CS. Acute pancreatitis: a multisystem disease. Gastroenterologist. 1993;1(2):115–28. 8049885

[2] Nadkarni NA, Khanna S, Vege SS. Splanchnic venous thrombosis and pancreatitis. Pancreas. 2013;42(6):924–31. 10.1097/MPA.0b013e318287cd3d 23587854

[3] Park WS, Kim HI, Jeon BJ, Kim SH, Lee SO. Should anticoagulants be administered for portal vein thrombosis associated with acute pancreatitis? World J Gastroenterol. 2012;18(42):6168–71. 10.3748/wjg.v18.i42.6168 PMC3496897 23155349

[4] Ahmed M, Aziz MU, Mansoor MA, Anwar S. Vascular complications in cases of acute pancreatitis - CT scan based study. J Pak Med Assoc. 2016;66(8):977–89. 27524531

[5] Deiss R, Young P, Yeh J, Reicher S. Pulmonary embolism and acute pancreatitis: case series and review. Turk J Gastroenterol. 2014;25(5):575–7. 10.5152/tjg.2014.6089 25417623

[6] Patel R, Choksi D, Chaubal A, Pipaliya N, Ingle M, Sawant P. Renal vein and inferior vena cava thrombosis: a rare extrasplanchnic complication of acute pancreatitis. ACG Case Rep J. 2016;3(4):e172. 10.14309/crj.2016.145 PMC5171928 28008405

[7] Mukund A, Gamanagatti S, Saraya A. Chronic pancreatitis causing thrombotic occlusion of IVC and renal veins. Trop Gastroenterol. 2011;32(4):337–8. 22696924

[8] Lee K, Ko JI, Park T. Acute pancreatitis complicated by massive inferior vena cava and right atrial thrombosis: a case report. Ann Vasc Surg. 2015;29(5):1020.e7–1020.e10. 10.1016/j.avsg.2015.01.019 25765632

[9] Arleo EK, Mennitt K. Celiac artery trunk thrombosis: an unusual complication of pancreatitis diagnosed on MRI. Clin Imaging. 2011;35(1):73–6. 10.1016/j.clinimag.2010.03.004 21237421

[10] Thajudeen B, Budhiraja P, Bracamonte ER. Bilateral renal artery thrombosis secondary to acute necrotizing pancreatitis. Clin Kidney J. 2013;6(5):503–6. 10.1093/ckj/sft106 PMC4438417 26064514

[11] Mallick IH, Winslet MC. Vascular complications of pancreatitis. JOP. 2004;5:328–37. 15365199

[12] Akbal E, Demirci S, Koçak E, Köklü S, Başar O, Tuna Y. Alterations of platelet function and coagulation parameters during acute pancreatitis. Blood Coagul Fibrinolysis. 2013;24(3):243–6. 10.1097/MBC.0b013e32835aef51 23425662

[13] Bradbury MS, Kavanagh PV, Bechtold RE, Chen MY, Ott DJ, Regan JD, *et al.* Mesenteric venous thrombosis: diagnosis and noninvasive imaging. Radiographics. 2002;22(3):527–41. 10.1148/radiographics.22.3.g02ma10527 12006685

[14] Moll S. Thrombophilia: clinical-practical aspects. J Thromb Thrombolysis. 2015;39(3):367–78. 10.1007/s11239-015-1197-3 25724822

[15] Ageno W, Beyer-Westendorf J, Garcia DA, Lazo-Langner A, McBane RD, Paciaroni M. Guidance for the management of venous thrombosis in unusual sites. J Thromb Thrombolysis. 2016;41(1):129–43. 10.1007/s11239-015-1308-1 PMC4715841 26780742

[16] Edwards L, Wanless IR. Mechanisms of liver involvement in systemic disease. Best Pract Res Clin Gastroenterol. 2013;27:471–83. 10.1016/j.bpg.2013.08.002 24090936

