# Supplementary material for: Association of multiple splanchnic venous thrombosis and left renal venous thrombosis, a rare complication of pancreatitis: a case report
Source: J Med Case Rep. 2019 Jun 4;13:171. doi: 10.1186/s13256-019-2053-4 (PMC6547583; doi:10.1186/s13256-019-2053-4)
Supplement: Supplementary file 1 — Relevant Past Medical History and Interventions (DOCX 15 kb) [file 13256_2019_2053_MOESM1_ESM.docx]

| Dates | **Relevant Past Medical History and Interventions** | | |
| --- | --- | --- | --- |
|  | - 48 years old patient - No medical history | | |
| Date | **Summaries from Initial and Follow-up Visits** | **Diagnostic Testing (including dates)** | **interventions** |
| **16juillet 2017** | - Patient admitted with epigastric pain and vomiting - Physical examination:   - Epiegastric tendress   - No jaundice   - No hepatic failure | - Contrast Enhanced Computed Tomography (16/07/2107):   - Balthazar grade C pancreatitis   - splanchnic thrombosis (portal vein, superior mesenteric vein)   - left renal vein thrombosis   - venous enteromesenteric infarct   - no signs of bowel perforation or effusion   - ischemic lesion in segment V,VI, VII and VIII - blood tests:   - lipase level: 600UI/l   - CRP: 28mg/   - renal function, hepatic function tests, coagulation and platelet without abnormalities - Thrombophilia screening (25/07/2017):   - Anti-DNA, Antinuclear and anticardiolipin antibodies, anti-B2GP1 and anti-FII were negative   - No abnormalities in activity of Antithrombin III, protein C and protein S - Viral serologies were negatives | - Anticoagulation: enoxaparin 1mg/kg - Analgesia: paracetamol+ nefopam - therapeutic measures for vimiting - resumption of enteral feeding after 72H |
| **2months later** | - patient admitted in the ICU for confusion - Physical examination :   - jaundice,   - mild tachycardia   - normal blood pressure   - hypoglycemia.   - Abdomen was distended, tense and tender on palpation   - ascites. | - CT abdomen:   - persistent splanchnic and renal thrombosis   - worsening of hepatic lesions with extension of bowel ischaemia.   - no signs of necrosis or bowel perforation - abnormalities in hepatic blood investigation:   - PT :25%,   - Aspartate Aminotransferase (AST) was 10 times normal,   - Alanine Aminotransferase (ALT) 8 times normal   - total bilirubin 180mg/l. | - stopping anticoagulation - therapeutic measures for hepatic failure - no specific therapeutic: thrombolysis or endovascular embolectomy |
| **48H later** | - grade 4 hepatic encephalopathy - multiple organ failure |  |  |
|  | - outcome: death |  |  |
